# Survival after out-of-hospital cardiac arrest during nights and weekends

**DOI:** 10.1186/cc10876

**Published:** 2012-03-20

**Authors:** K Maekawa, K Tanno, M Hase, K Mori, Y Asai

**Affiliations:** 1 Sapporo Medical University, Sapporo, Japan

## Introduction

Out-of-hospital cardiac arrest (OOHCA) still has a low survival rate, despite considerable efforts including early applications of basic life support and defibrillation in the pre-hospital setting. Post-resuscitation care after hospitalization, influencing the final outcome, may be less available during nights and weekends because of hospital, staffing, and response factors. We sought to determine whether outcomes after OOHCA differ during nights and weekends (off-hours) compared with daytimes of weekdays (on-hours).

## Methods

We performed a retrospective analysis of 4-year data collected prospectively in a single institute. Adults with witnessed OOHCA of cardiac origin were recruited. The therapeutic strategy after hospitalization, including extracorporeal cardiopulmonary resuscitation (ECPR), therapeutic hypothermia (TH) and primary percutaneous coronary intervention (PCI), was dependent on the critical care physicians in charge. We used a propensity-score matching to reduce the differences of pre-hospital variables between patients arriving during off-hours and on-hours. Primary endpoint was 90-day survival after cardiac arrest. We evaluated the survival difference using the log-rank test and identified the significant interventions affecting outcome using the Cox regression model.

## Results

Of 185 patients, 131 arrived during off-hours (the off-hours group) and 54 arrived during on-hours (the on-hours group). The matching process selected 37 patients each from both groups. The matched off-hours group had a lower survival rate than the matched on-hours group (10.8% vs. 37.8%; log-rank *P *= 0.025). Multivariate Cox regression analysis showed that TH was associated with 90-day survival after cardiac arrest (adjusted hazard ratio (HR), 0.43; 95% CI, 0.23 to 0.79), but there were no significant associations of ECPR (adjusted HR, 0.83; 95% CI, 0.50 to 1.37) and primary PCI (adjusted HR, 0.76; 95% CI, 0.42 to 1.38).

## Conclusion

Lower survival rates after OOHCA during nights and weekends were seen at our institute. TH was more likely to be induced in patients arrived during daytimes of weekdays, and independently associated with survival benefit.
